# Maintaining Genome Integrity: Protein Kinases and Phosphatases Orchestrate the Balancing Act of DNA Double-Strand Breaks Repair in Cancer

**DOI:** 10.3390/ijms241210212

**Published:** 2023-06-16

**Authors:** Sisi Qin, Ichiwa Kitty, Yalan Hao, Fei Zhao, Wootae Kim

**Affiliations:** 1 Department of Pathology, University of Chicago, Chicago, IL 60637, USA; sisiq@bsd.uchicago.edu; 2 Department of Integrated Biomedical Science, Soonchunhyang Institute of Medi-Bio Science (SIMS), Soonchunhyang University, Cheonan 31151, Chungcheongnam-do, Republic of Korea; kitty.201908510004@student.atmajaya.ac.id; 3 Analytical Instrumentation Center, Hunan University, Changsha 410082, China; haoyalan99@outlook.com; 4 College of Biology, Hunan University, Changsha 410082, China

**Keywords:** DNA damage, cancer therapeutics, kinase, phosphatase

## Abstract

DNA double-strand breaks (DSBs) are the most lethal DNA damages which lead to severe genome instability. Phosphorylation is one of the most important protein post-translation modifications involved in DSBs repair regulation. Kinases and phosphatases play coordinating roles in DSB repair by phosphorylating and dephosphorylating various proteins. Recent research has shed light on the importance of maintaining a balance between kinase and phosphatase activities in DSB repair. The interplay between kinases and phosphatases plays an important role in regulating DNA-repair processes, and alterations in their activity can lead to genomic instability and disease. Therefore, study on the function of kinases and phosphatases in DSBs repair is essential for understanding their roles in cancer development and therapeutics. In this review, we summarize the current knowledge of kinases and phosphatases in DSBs repair regulation and highlight the advancements in the development of cancer therapies targeting kinases or phosphatases in DSBs repair pathways. In conclusion, understanding the balance of kinase and phosphatase activities in DSBs repair provides opportunities for the development of novel cancer therapeutics.

## 1. Introduction

DNA exposed to radiation or chemical agents may cause damages. The resulting damages lead to a higher occurrence of mutations and genome instability, eventually leading to the development of various diseases, including cancer. However, mammalian cells have evolved several mechanisms to detect and repair those DNA damages. The DNA-repair signaling is activated immediately when DNA damages are detected, resulting in the activation of cell-cycle checkpoint and recruitment of DNA-repair proteins to damage sites [1].

Phosphorylation is an important protein post-translational modification that regulates DNA-repair signaling [2]. Enzymes can be divided into two categories, kinases and phosphatases, both of which are involved in maintaining the regulation of protein phosphorylation during DNA damage. These enzymes are responsible for the reversible phosphorylation of proteins. Kinases add a phosphate group to specific amino acids on proteins, while phosphatases remove the phosphate group. The interplay between kinases and phosphatases enables cells to rapidly and precisely control a broad range of cellular processes, including DNA repair.

Numerous kinases and phosphatases have been proved to have function on DNA repair. These enzymes are responsible for modulating DNA damage response (DDR), controlling the cell cycle, and acting in a coordinated manner to regulate DNA-repair processes [3]. Briefly, most kinases play roles in initiating and modulating DNA repair under different types of DNA damage, including DSBs, SSBs (singledouble-strand breaks), stalled replication forks, or UV-induced base damages. Those kinases function by phosphorylating a broad range of substrates involved in the DNA-repair pathway. ATM (Ataxia-Telangiectasia Mutated), ATR (ATM and RAD3-related), and DNA-PK (DNA-dependent protein kinase), the three most important kinases in DNA repair, work together or respectively to activate downstream kinases, such as CHK1 (checkpoint kinases 1) and CHK2 (checkpoint kinases 1), which, in turn, phosphorylate other downstream targets [4]. Phosphatases function in opposition to kinases by removing phosphate groups from DNA-repair-related proteins. These enzymes play an essential role in terminating kinase signaling, and are required for resetting the DNA-repair machinery. For example, PP1 (protein phosphatase 1) or WIP1 phosphatases terminate DNA damage response by dephosphorylating various ATM and Chk2 substrates [5].

The balance between kinase and phosphatase activities is important for proper DDR signaling transduction. Alterations in kinase or phosphatase activity lead to genome instability. For example, ATM mutation leads to reduced DNA-repair efficacy and accumulation of double-strand breaks in cells, resulting in higher mutation frequency and genome instability. Inactivating mutations in PP1, Wip1, or other phosphatases can lead to hyperactivation of DDR signaling and a predisposition to cancer [5].

In conclusion, kinases and phosphatases function coordinately in DNA repair and maintaining genome stability. Understanding the functions of kinases and phosphatases in DNA repair will enable the development of targeted therapies to improve the therapeutics for diseases such as cancer.

## 2. Kinases in the DNA DSBs Repair

In mammalian cells, protein phosphorylation kinases are crucial components in the DSB signaling cascade, from sensing DNA damage, to transducing signals, to repairing DSBs. The protein kinases are traditionally categorized as either apical or effector kinases [4,6]. Most of the protein kinases involved in DNA DSB repair are evolutionarily conserv ed, from yeast to mammalian systems. To date, facilitated by mass spectrometry (MS)-based proteomics, dozens of DNA-repair proteins have been identified to be phosphorylated by these kinases and a more systematic network of phosphorylation events triggered by kinases has been established [6,7,8]. Therefore, we have gathered a more comprehensive vision of the machinery of the DSBs repair. 

### 2.1. Apical (Sensor) Kinases

**Figure 1:**
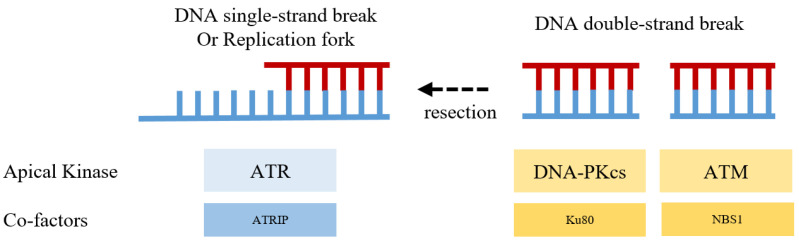
The Apical (sensor) kinases and co-factors in DNA double-strand break repair response.

The apical kinases in DSB repair, including DNA-PKcs (DNA-dependent protein kinase, catalytic subunit), ATM, and ATR, belong to the superfamily of phosphatidylinositol-3-kinase-related kinases (PIKKs) [4,9]. DNA-PKcs, ATM, and ATR are serine/threonine protein kinases, and, as PIKKs members, they share some structural similarity in the catalytic loop within the kinase domain [9]. DNA-PKcs, ATM, and ATR can be activated by DSBs and, once activated by DSBs, they lie at the heart of the signal-transduction process as they can phosphorylate a broadboard and overlapping spectrum of substrates transducing DNA damage signals (Figure 1) [9,10]. 

There are two pathways that dominate the repair of DSBs: non-homologous end-joining (NHEJ) and homologous recombination (HR) [11,12,13,14]. NHEJ repairs most DSBs in mammalian cells, while HR is preferred when NHEJ cannot be completed [15,16]. DNA-PK is a critical kinase in the NHEJ pathway [4]. ATM is essential for the initiation and completion of the DSB repair by HR pathway [17,18,19]. ATR is activated in response to persistent single-stranded DNA, which could be an intermediate in DSB repair pathways such as HR [20,21].

In response to DSBs and being tightly regulated by their co-factors (Ku80 for DNA-PKcs, NBS1 for ATM, and ATRIP for ATR) [22,23,24], the apical kinases can be activated by auto-phosphorylation [4]. Once activated, they can phosphorylate H2A histone family member X (H2AX). The pS139 of H2AX (γH2AX), a marker for DSBs, generates binding sites for mediators/adaptors proteins such as DNA damage checkpoint protein 1 (MDC1), breast cancer susceptibility protein 1 (BRCA1) and p53 binding protein 1 (53BP1) [10,25,26]. The apical kinases then transfer the phosphorylation to downstream effector kinases via mediators in the DSB repair pathways [4].

Specifically, DNA-PKcs can be auto-phosphorylated or occasionally phosphorylated by ATM and ATR [27,28]. S2056 and T2609 are two major phosphorylation sites of DNA-PKcs, and both are crucial for its activity in DNA repair [8]. Once activated, DNA-PKcs dissociates from the DSBs and phosphorylates a number of substrates, including Replication protein A (RPA), Werner syndrome ATP-dependent helicase (WRN), Artemis, and H2AX [8,29,30]. 

Upon DNA damage, ATM auto-phosphorylates on S1981, then further auto-phosphorylates on S367, S1893, S1981, and, potentially, other sites to activate the ATM kinase [31,32]. Proteins involved in the ATM signaling, such as CHK2, p53, BRCA1, NBS1, and H2AX, are all targets of activated ATM [33].

**Figure 2:**
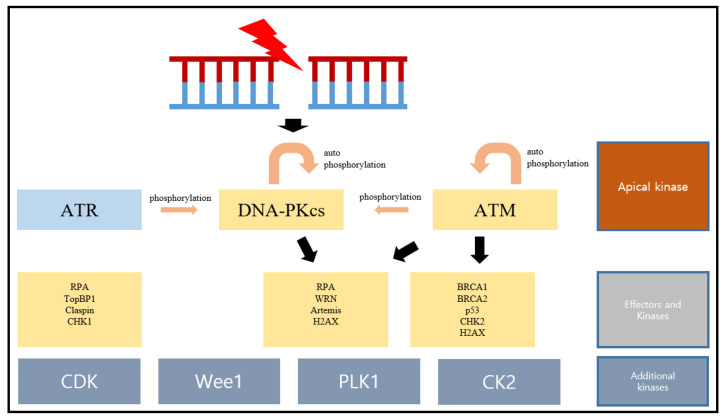
The targets of kinases in DNA double-strand break repair response.

Unlike DNA-PKcs and ATM, ATR is activated by DSBs and also a broad spectrum of other DNA damages [20]. Autophosphorylation T1989 is critical for ATR activation [34]. Many of the proteins in ATR signaling network, such as RPA, TopBP1 (DNA topoisomerase II binding protein 1), Claspin, and CHK1, are substrates of ATR [20] (Figure 2). 

### 2.2. Effector Kinases

In mammalian cells, activated apical kinases transfer stimulatory phosphorylation to the downstream checkpoint kinases CHK1 and CHK2. As shown in Figure 2, in response to DSBs, CHK2 is activated via phosphorylation on T68 by ATM and, subsequently, oligomerization and autophosphorylation at T383 and T387 [35,36,37], while, in response to single-stranded DNA which can be generated from DSBs, CHK1 is phosphorylated on S317 and S345 by ATR kinase to become activated [38]. Both CHK1 and CHK2 are serine/threonine-specific protein kinases. Activated CHK1 and CHK2 phosphorylate a variety of downstream substrates, such as p53 and CDC25A (cell division cycle 25A), to activate DNA-repair pathways, bring out cell-cycle arrest, or induce apoptosis when DNA damage is severe [39,40].

### 2.3. Additional Kinases

There are other kinases involved in the DSB repair downstream signaling, such as cyclin-dependent kinases (CDKs), WEE1, polo-like kinase 1 (PLK1), and casein kinase II (CK2); see Figure. 2. They can be phosphorylated by apical and effector kinases. They also belong to the serine/threonine family of protein kinases and play important roles in repairing DNA and regulating a range of other processes, such as the cell-cycle arrest and initiation of apoptosis [6]. 

In addition to the serine/threonine kinases mentioned above, a number of tyrosine kinases have been demonstrated to participate in the DSBs repair signaling pathways, as previously described [41]. In brief, tyrosine kinases, such as epidermal growth factor receptor (EGFR), insulin-like growth factor 1 receptor (IGF-1R), ACK1/ TNK2, and Src family of tyrosine kinases, can be activated by DNA damage and epigenetically regulate DNA damage signaling by phosphorylating the core histones and chromatin modifiers [41]. 

### 2.4. Kinase Mutations and Related Diseases

As DNA damage response can be activated at early stages of tumorigenesis, the protein kinases involved in the DNA-damage-signaling pathways may serve as a barrier to tumorigenesis and development [42,43]. Concordant with this, the key kinase genes in DSB repair, ATM and ATR, are frequently mutated in various human cancers [44]. The XRCC7 or PRKDC gene (which encodes DNA-PK) has also been reported to present a higher mutation rate in certain types of cancers, such as colorectal, gastric, and endometrial cancers [45]. The CHK2 gene has also been found to be associated with some types of cancers, such as breast, kidney, and thyroid cancers [46]. The CHK1 gene, however, is not regarded as a canonical tumor suppressor based on its conflicting evidence in cancer [47]. 

Actually, most of the disease related to aberrations in DSBs repair protein kinases are marked by growth and developmental problems, such as cancer (abnormal cell proliferation, see details above), Ataxia telangiectasia (with immune and neuron dysfunctions, and radiosensitivity and cancer predisposition features; associated with ATM, PRKDC, CHK1, or CHK2 mutations) [48,49] and Seckel syndrome (growth retardation; associated with ATR mutations) [50].

In addition, the kinase-regulated DNA repair is crucial for the generation of diverse foreign antigen. The failure to process DNA repair can lead to immunodeficiency disease [51], such as Ataxia telangiectasia (mentioned above) and severe combined immunodeficiency (associated with PRKDC mutations) [52]. 

Regarding the mutation hotspots and mutation types in cancer, most of the truncating mutations of ATM, ATR, CHEK1, and CHEK2 are considered to be, at least, likely pathogenic. While missense mutations in ATR and CHK1 are not common, ATM missense mutations have been reported across the whole ATM gene. Moreover, pathogenic/likely pathogenic missense mutations of ATM and CHK2 are mainly located within their kinase domains [53,54,55]. 

## 3. Phosphatases in DSBs Repair

Though the activation of DSBs-repair signaling is driven by the phosphorylation cascades of DNA-repair proteins, the steady state of phosphorylation is regulated by both kinases and phosphatases. Therefore, reversible phosphorylation of DNA-repair proteins is an important regulatory mechanism in DSBs repair [53,54,55]. 

Unlike kinases that share a common catalytic fold and mechanism, phosphatases present greater diversity of structures and catalytic mechanism [56]. Thus, based on the differences in sequence, structure, and catalytic mechanisms, protein phosphatases that are involved in DSBs repair can be divided into different groups: the classic serine/threonine phosphoprotein phosphatase (PPP) family (comprising PP1, PP2A, PP2B, PP4, PP5, PP6, and PP7), the serine/threonine protein phosphatase family that is Mg2+ or Mn2+-dependent (PPM) (including PP2C), and the dual-specificity phosphatase (DUSP) family (such as cyclin-dependent kinase (CDK)-antagonizing cell-division cycle 14 (CDC14)) [5,57].

### 3.1. PPP Family

Among the PPP family members, PP1, PP2A, PP2B, PP4, and PP6 are all considered as split enzymes, as their selective dephosphorylation function requires the assembly of two modules, the catalytic subunit and the regulatory (non-catalytic) subunit [56]. 

PP1 (protein phosphatase 1) is an abundant protein in cells and is responsible for dephosphorylation of about one third of all phosphor-proteins [5,56]. The PP1 phosphatase has been demonstrated as a main regulator in DNA repair. Repo-Man/PP1γ holoenzyme (with Repo-Man as the regulatory component) can dephosphorylate ATM at S1981 and reduce ATM kinase activity [58]. Together with PP1-binding factor PNUTS (phosphatase 1 nuclear targeting subunit), Repo-Man/PP1 can also regulate the phosphorylation status of CHK1, H2AX, 53BP1, RPA, and RAD51 [59,60]. 

The conserved region of the catalytic subunit of PP2A is similar to that of PP1, which may explain the overlapping targets (including ATM, CHK1, γ-H2AX, and RPA) of these two phosphatases in DNA repair [57]. The distinguished wide range of regulatory subunit of PP2A also provide the holoenzyme with the ability to recognize and mediate other proteins in DNA repair, such as DNA-PK and CHK2 [57,61]. 

PP2B is also called PP3 or calcineurin. Little is known of its involvement in the direct dephosphorylation of DNA-repair proteins. PP4 is 41% identical to PP1 and 65% identical to PP2A in human. PP4 is a ubiquitously expressed phosphatase and it regulates multiple cellular processes, including DNA repair independently from PP1 or PP2A. In humans, PP4 is capable of dephosphorylating γ-H2AX and RPA [57,62]. 

Different from other PPP family members, PP5 is a single subunit enzyme [63]. In mammalian cells, PP5 has been shown to interact with ATM and ATR in response to DNA damage. PP5′s interaction with ATM/ATR is necessary for ATM/ATR activation, and DNA-PK can be dephosphorylated by PP5 at T2609 to modulate the DNA-PK signaling [64]. Besides, there is also a reciprocal regulatory interplay between p53 and PP5, as PP5 can directly dephosphorylate p53 while p53 suppresses PP5′s transcription [65].

Though closely related to PP2A and PP4, PP6 has not been well studied. Some evidence showed that PP6 is recruited to DSBs sites by DNA-PK and PP6 may dephosphorylate γ-H2AX [66,67]. PP7 can target various phosphoproteins, such as myelin basic protein. Its function in DNA repair is not clearly known.

### 3.2. PPM Family

In contrast to the PPP family, PPM phosphatases are mostly monomeric enzymes [65]. PPM family members all share a highly similar catalytic core and they are resistant to classic inhibitors and toxins of the PPP family [54]. PP2C belongs to the PPM family and among the PP2C family members, WIP1/PPM1D (wild-type p53-inducible phosphatase 1) has been shown to actively participate in DNA repair. In human cells, WIP1 dephosphorylates CHK1, CHK2, and p53 to downregulate DNA-damage checkpoint signaling and enhance cell-cycle recovery [68,69,70]. It can dephosphorylate γ-H2AX to inhibit the activation of the DNA-damage checkpoint. Moreover, Wip1 can dephosphorylate Atm at S1981 to modulate Atm activation in mice [71,72]. 

Another PP2C phosphatase, PPM1G (also termed PP2Cγ), also has a role in the DNA repair. It can mediate the exchange of H2A-H2B, and PPM1G-deficient cells are sensitive to DNA damage. It is a target of ATM and dephosphorylates USP7 (ubiquitin specific peptidase 7) to regulate p53 [5,73].

### 3.3. DUSP Family

Within the DUSPs, CDC14, which is capable of dephosphorylating both phosphoserine/phosphothreonine and phosphotyrosine residues, has been involved in DNA repair. Several knockout experiments conducted in mammalian cells have implied the direct role of CDC14A and CDC14B in DSBs repair [57]. CDC14 tends to dephosphorylate substrates that are previously phosphorylated by CDK, suggesting its role counteracting CDK substrates in the DNA repair process [57,62]. 

### 3.4. Phosphatase Alterations and Related Diseases

Other than involving in the DNA repair, protein phosphatases have a broad substrate specificity and diverse cellular functions, including cell division, apoptosis, protein synthesis, glycogen metabolism, neuronal activities, etc. Therefore, aberrations of phosphatases are related to a variety of diseases, such as cancers, viral infection, and heart diseases [74,75,76,77].

Compared to kinases, DNA-damage-repair-related phosphatase genes are less frequently mutated, their differential expression and interaction alterations between the substrates and phosphatases can also result in cellular dysfunction and disorders [78,79].

## 4. Dynamic Balance between Kinases and Phosphatases in DSBs Repair and Cancer Therapeutics

Upon DNA DSBs, ATM, ATR, and DNA-PK initiate signaling transduction. These kinases then activate CHK1 and CHK2 kinases and other substrates to result in DNA repair or apoptosis [3]. DNA-repair signaling is relayed by protein phosphorylation cascades, and the protein phosphatases can negatively regulate these phosphorylation events [80]. Though most of the phosphatase negatively regulates the proteins in the DNA DSBs repair, certain phosphatases could enhance the activity of proteins in the pathway [80]. Table 1 is a summary of kinases, phosphatases, and their substrates in DSBs repair. The dynamic balance between kinases and phosphatases fine-tunes the phosphorylation cascades in the DNA-repair process, including regulating the DNA-damage checkpoint and the repair of DNA lesion [57].

Targeted therapy is a more precise and effective approach to treating cancer by delivering therapeutic agents specifically to cancer cells while minimizing harm to healthy ones. Researchers have developed inhibitors that target specific proteins involved in DSBs repair and cell-cycle regulation, such as ATM, ATR, DNA-PK, CHK1, CHK2, PP1, PP2A, and PP1D. By inhibiting these key kinases and phosphatases, the effectiveness of cancer therapy can be enhanced, rendering cancer cells more vulnerable to treatment-induced DNA damage [323,324]. Table 2 provides a detailed overview of specific inhibitors and their targets, further highlighting their potential in enhancing cancer therapy.

### 4.1. ATM Inhibitors

AZD0156 is an orally bioavailable ATM inhibitor that has been identified to interact with catalytic lysine (Lys2717), kinase hinge (Cys2770), and back pocket (Tyr2755) to bind with the ATP site of ATM. With a potent inhibition effect, AZD0156 can act as a radiosensitizer, inhibiting tumor growth, and promoting a killing effect on cancer cells. It can also be used in combination with some drugs, such as olaparib, fluorouracil, and irinotecan [325,326,327]. Previous research showed that AZD0156 could prevent the repair mechanism induced by olaparib-induced DNA damage, resulting in apoptotic cellular responses. However, as a single agent, AZD0156 has shown limited effects on colorectal cancer cells. The combination between AZD0156 and irinotecan can improve dsDNA breaks, leading to cell-cycle arrest [328].

Another ATM inhibitor, M3541, is an ATP-selective inhibitor that can impede DSB-repair mechanisms. With an IC50 less than 1 nM, it can increase the sensitivity of cancer cells and improve anti-tumor effects of ionizing radiation. The administration of M3541 orally in nude mice has also shown a decrease in tumor size [325,329,330]. The combination of an ATM inhibitor with a CHK2 inhibitor (NCT03571438) has been under investigation as a potential therapy leading to apoptosis [331]. On the other hand, there are some ATM inhibitors with IC50 higher than that of AZD0156 and M3541, such as KU-59403 (IC50 = 3 nM), KU60019 (IC50 = 6.3 nM), KU-55933 (IC50 = 13 nM), and AZ31 (IC50 = 46 nM) [327].

### 4.2. ATR Inhibitors 

Berzosertib, also known as VE-822, M6620, or VX-970, is a selective ATR inhibitor with an IC50 of 19 nM and has entered in clinical trial NCT04052555. It is an intravenously administered upgraded version of VE-822. Berzosertib can prevent the phosphorylation of γ-H2AX, which blocks DNA repair process and triggers DNA damage accumulation. Additionally, the combination of berzosertib with gemcitabine (NCT02157792) was found to be well-tolerated by patients. A previous study conducted on 48 patients showed that four patients had a partial response (PR), and 29 patients experienced no change. [332,333]. 

Ceralasertib, or AZD6738, is a potent and selective oral ATR inhibitor. The mechanism of ceralasertib is to impede the CHK1 phosphorylation and promote the γ-H2AX phosphorylation during stress response in the S-phase of the cell cycle [334]. Ceralasertib can induce an ATM-dependent signaling pathway and inhibit the HR-repair pathway. A previous study showed that anti-tumor activity and a decrease in tumor size was observed in patients treated with ceralasertib in combination with carboplatin (NCT02264678) [335]. Another study also described a combination of ceralasertib with durvalumab (NCT03780608) in gastric-cancer patients. This kind of combination can result in increased anti-tumor activity and loss of essential genes in DDR [336]. 

### 4.3. DNA-PK Inhibitor

Peposertib, or M3841, has been known as a DNA-PK inhibitor both in monotherapy and in combination with another agent. Peposertib is an orally selective inhibitor at sub-nanomolar concentrations. This inhibitor can impede DNA-PK and then trigger p53-dependent anti-tumor activity [337]. The selectivity of peposertib has been examined among several protein members of the phosphoinositide-3 kinase (PI3K) and PI3K-related family, such as ATR, ATM, and DNA-PK. As a result, the inhibition of peposertib on DNA-PK is significantly higher than that on other proteins. A previous clinical trial (NCT02316197) demonstrated that peposertib could be tolerated by patients and has displayed acceptable efficacy with the recommended phase 2 dose (RP2D) of 300 mg twice daily (BID) [338]. 

AZD7648 also has been discovered as a potent and selective DNA-PK inhibitor. AZD7648 is an orally administered inhibitor that can bind with the catalytic subunit of DNA-PK. Based on the previous study (NCT03907969), adding AZD7648 can improve the effectiveness of other anti-cancer agents, such as radiation and olaparib (PARP inhibitor). Compared to monotherapy treatment with AZD7648, radiation, or olaparib, combination treatment with AZD7648 can improve the inhibition of cancer growth, as well as reduce macronuclei formation and chromosomal aberration. [339,340].

### 4.4. CHEK1 and CHEK2 Inhibitors

LY2606368, known as prexasertib, is studied as a selective and ATP-competitive CHK1 inhibitor that can be used as a monotherapy or in combination with other inhibitors. LY2606368 is a second generation of CHK1 inhibitor with IC50<1 nM, while the first generation, such as LY2603618, has higher IC50 (about 8 nM). Not only does prexasertib inhibit CHK1, but it also inhibits CHK2, although the inhibitory effect is lower for CHK2. A previous study (NCT02808650) has demonstrated that prexasertib can induce apoptosis as a single agent [341,342,343]. Prexasertib can also be combined with PD-L1 antibody LY3300054 (NCT03495323) to activate the cytotoxic T-cell in Cyclin E1 (CCNE1)-amplified high-grade serous ovarian-cancer (HGSOC) patients [344]. 

MK-8776 is also known as a specific CHK1 inhibitor. It can act as a single agent or in combination with other drugs, such as gemcitabine and cytarabine (NCT00779584). MK-8776 was previously known as SCH 900776, a pyrazolo[1,5-a]pyrimidine derivative that binds with the hinge region located in the kinase ATP-binding site [341,345]. Previous studies show that MK-8776 treatment can enhance cancer-cell sensitivity to hydroxyurea, gemcitabine, and radiation, leading to DNA damage accumulation [346,347].

On the other hand, CHK2 inhibitors are less effective in cancer treatment. PHI-101 (NCT04678102) was discovered as an orally administered and highly selective CHK2 inhibitor with anti-tumor activity in ovarian cancer. It impedes the DSBs repair pathway in cancer, especially in platinum-resistant recurrent ovarian cancer [348]. CCT241533, containing a 2-(quinazolin-2-yl) phenol scaffold, is another highly selective inhibitor of CHK2, and is also a moderate selective inhibitor of CHK1. CCT241533 can be used as a single agent or combined with chemotherapy drugs, such as topotecan, camptothecin, and etoposide for cancer therapy [349].

### 4.5. PP1 Inhibitors

Different types of PP1 inhibitors have been identified, targeting either the catalytic site or the regulatory subunits of PP1. Natural product PP1 inhibitors include okadaic acid, microcystin-LR, cantharidin, and tautomycin, which have been isolated from marine organisms, cyanobacteria, and higher plants (1–2). These compounds bind to the catalytic subunit of PP1 and prevent it from dephosphorylating its substrates. On the other hand, synthetic PP1 inhibitors have been designed and synthesized based on the structure of natural product PP1 inhibitors. These inhibitors can be further divided into several structural classes, such as indole derivatives, benzimidazole derivatives, and pyridine derivatives. 

PP1 inhibitors have shown promise in preclinical studies as potential therapeutics for cancers. For instance, fostriecin has demonstrated antitumor activity in animal models of human cancer (3). However, the development of PP1 inhibitors as clinical therapeutics is still in its early stages, and more research is needed to optimize their efficacy and safety.

### 4.6. PP2A Inhibitors

LB100, also known as LB1, has been recorded as a norcantharidin derivative compound, while norcantharidin was known as a traditional PP2A inhibitor. LB100 can bind and competitively inhibit the PP2A protein, which can sensitize cells to chemotherapy and radiotherapy. A phase I clinical trial of LB100 (NCT01837667) reported that LB100 has anti-tumor activity alone and is safe and tolerated by adult patients. In addition, a phase II clinical trial of LB100 (NCT03027388) showed an anti-glioblastoma (GBM) effect from LB100 in combination with PRMT5 inhibition. LB100 has also been used in combination therapy with doxorubicin or temozolomide as a DNA-damage agent, leading to the inhibition of cancer-cell growth. [350,351,352,353].

### 4.7. PPM1D Inhibitors

GSK2830371 is a potent and selective allosteric WIP1/PPM1D inhibitor used in cancer therapy. Its binding with the flap subdomain of the protein can confer significant effects on cell death and sensitize cells to chemotherapy. GSK2830371 can also reduce the doxorubicin dose, alleviating side-effects. Furthermore, GSK2830371 can stimulate the inhibition of cell proliferation and lead to cell-cycle arrest. [354,355,356,357].

**Table 2:** Cancer therapeutic inhibitors and their targets, analyzed based on clinicaltrials.gov database (accessed on 21 April 2023).

Target	National Clinical Trials Number	Clinical Phase	Disease	Treatment	Title	Result
ATM	NCT02588105	Phase 1	Advanced solid tumors	Drug: AZD0156 Drug: Olaparib Drug: Irinotecan Drug: Fluorouracil Drug: Folinic acid	Study to assess the safety and preliminary efficacy of AZD0156 at increasing doses alone or in combination with other anti-cancer treatment in patients with advanced cancer (AToM)	Increased radiosensitivity [325], increased anti-tumor activity of olapararib [326], enhanced DSBs [328]
	NCT03225105	Phase 1	Solid tumors	Drug: M3541 Radiation: Palliative radiotherapy (RT)	M3541 in combination with radiotherapy in subjects with solid tumors	Decreased the tumor size, radiosensitivity [329,330], apoptosis [331]
ATR	NCT04052555	Phase 1	Bilateral breast carcinoma HER2-negative breast carcinoma Localized breast carcinoma Recurrent breast carcinoma Triple-negative breast carcinoma	Drug: Berzosertib Procedure: biospecimen collection Other: quality of life assessment Other: questionnaire administration Radiation: radiation therapy	Testing the addition of an anti-cancer drug, berzosertib, to the usual treatment (radiation therapy) for chemotherapy-resistant triple-negative and estrogen and/or progesterone receptor positive, HER2 negative breast cancer	Blocked DNA repair process, accumulated DNA damage [333]
	NCT02157792	Phase 1	Advanced solid tumor	Drug: M6620	M6620 first in human study	RP2D is berzosertib 210 mg/m^2^ for days 2 and 9 + gemcitabine 1000 mg/m^2^ for days 1 and 8 within 3 weeks [332]
	NCT02264678	Phase 1	Advanced solid Malig—H&N SCC, ATM Pro/Def NSCLC, gastric, breast and ovarian cancer	Drug: Gemcitabine	Ascending doses of ceralasertib in combination with chemotherapy and/or novel anti-cancer agents	RP2D for combination with carboplatin : ceralasertib 40 mg for days 1 and 2 + carboplatin every 3 weeks [335]
	NCT03780608	Phase 2	Gastric adenocarcinoma	Drug: Cisplatin	This study is a phase II study of AZD6738 in combination with durvalumab in patients with solid tumor (cohort A (N = 30): GC who have failed secondary chemotherapy treatments regimen; cohort B (B = 30): Melanoma patients who have failed immunotherapy (IO))	Increased anti-tumor activity [336]
DNA-PK	NCT02316197	Phase 1	Advanced solid tumors Chronic lymphocytic leukemia	Drug: MSC2490484A (M3814)	Clinical phase I study investigating MSC2490484A, an inhibitor of a DNA-dependent protein kinase, in advanced solid tumors or chronic lymphocytic leukemia	RP2D is 400 mg of peposertib [338]
	NCT03907969	Phase 1 Phase 2	Advanced malignancies	Drug: AZD7648 Drug: PLD	A clinical trial to evaluate AZD7648 alone and in combination with other anti-cancer agents in patients with advanced cancers	Inhibited cancer growth, increased macronuclei formation, and chromosomal aberration [339]
CHK1	NCT03495323	Phase 1	Cancer	Drug: LY3300054 Drug: Prexasertib	A study of prexasertib (LY2606368), CHK1 inhibitor, and LY3300054, PD-L1 inhibitor, in patients with advanced solid tumors	Activated the T-cells in blood samples of CCNE1-amplified HGSOC patients [344]
	NCT02808650	Phase 1	Childhood solid neoplasm Recurrent malignant solid neoplasm Recurrent primary central nervous system neoplasm Refractory malignant solid neoplasm Refractory primary central nervous system neoplasm	Other: Laboratory biomarker analysis Other: Pharmacological study Drug: Prexasertib	Prexasertib in treating pediatric patients with recurrent or r efractory solid tumors	Apoptosis, 150 mg/m^2^ administered IV for day 1 and day 15 of 28 days [341,342]
	NCT00779584	Phase 1	Hodgkin’s disease Lymphoma, Non-Hodgkin’s neoplasms	Drug: MK-8776 Drug: Gemcitabine	A dose-escalation study of MK-8776 (SCH 900776) with and without gemcitabine in participants with solid tumors or lymphoma (MK-8776-002/P05248)	RP2D is 200 mg/m^2^for MK-8776 with gemcitabine (1000 mg/m^2^) for day 1 and 8 of 21 days [347]
CHK2	NCT04678102	Phase 1	Platinum-resistant ovarian cancer Platinum-refractory ovarian carcinoma Platinum-resistant fallopian tube carcinoma Platinum-resistant primary peritoneal carcinoma	Drug: PHI-101 administration	CHK2 inhibitor for Recurrent EpitheliAl periToneal, fallopIan, or oVarian cancEr (CREATIVE Phase IA Trial)	Inhibited DNA repair, provided anti-tumor activity [349]
PP2A	NCT01837667	Phase 1	Tumors Neoplasms Cancer	Drug: LB-100 for injection Drug: Docetaxel	Phase I study of LB-100 with Docetaxel in solid tumors	RP2D is 2.33 mg/m^2^ for 3 consecutive days every 3 weeks [351]
	NCT03027388	Phase 2	Astrocytoma Grades II, III, and IV Glioblastoma Multiforme Giant cell Glioblastoma Glioma Oligodendrogliomas	Drug: LB-100	Protein phosphatase 2A inhibitor, in recurrent glioblastoma	Inhibited cancer cells growth [352]

## 5. Challenge and Perspective

Kinases and phosphatases have essential function on coordinating DNA-repair pathways. Dysregulation of these enzymes leads to enhanced genomic instability, which is a hallmark of cancer. Understanding the functions of kinases and phosphatases in DNA-repair regulation is essential for developing effective cancer therapeutics.

Kinases and phosphatases work in opposition to each other, and the balance between their activities is critical for proper DDR signaling and cancer-cell survival. Therefore, developing inhibitors to modulate kinase or phosphatase activity to disrupt DDR signaling is a promising strategy for cancer therapeutics. However, one of the main challenges in studying kinases and phosphatases in DNA repair is identifying specific targets for therapeutic intervention. There are numerous kinases and phosphatases involved in DNA repair. However, many of these enzymes have multiple functions and substrates. For example, ATM is an essential kinase involved in DNA repair, but it also plays roles in other cellular processes, such as RNA transcription or energy metabolism which also affect the survival of normal cells. The safety of these inhibitors should be intensively evaluated in clinical trials. Therefore, developing kinases and phosphatases inhibitors that specifically target the DNA-repair pathway but with fewer side-effects on other signaling is important. Another challenge is that inhibitors of DNA-repair-related kinases and phosphatases have shown efficacy in treating certain types of cancer, whereas their use has not been approved for all types of cancer in clinical trials. Therefore, identifying biomarkers that can predict response to the inhibitors of DNA-repair-related kinases or phosphatases will help clinicians to identify which groups of patients are more likely to respond to these inhibitors. Predictive biomarkers can help optimize treatment strategies and improve patient outcomes. The use of deep sequencing approaches to identify specific genetic alterations in cancer cells may provide opportunities for precision therapies that selectively target kinases or phosphatases in DNA-repair pathways.

Despite these challenges, recent studies have provided several promising options for the development of cancer therapeutics targeting kinases and phosphatases in DNA repair. Multiple inhibitors targeting kinases or phosphatases involved in DNA repair have shown promise in pre-clinical studies or clinical trials which may provide us with alternative choices in the toolbox for cancer therapeutics. Besides, the combination of traditional radio-therapy or chemo-therapy with kinase or phosphatase inhibitors is also a potential strategy for cancer treatment. Cancer cells frequently develop resistance to radio-therapy or single-agent chemo-therapies by acquiring altered DNA-repair activity. Therefore, a combination treatment with kinase or phosphatase inhibitors may disrupt the DNA-repair ability of cancer cells under radio- or chemo-therapy and may enhance therapeutics efficacy.

In conclusion, studies on kinases and phosphatases in DNA repair are rapidly expanding fields that provide valuable insights into the mechanisms involved in genome stability, cancer development, and cancer therapeutics. While there are still significant challenges and limitations on developing targeted therapeutics, further studies on the development of novel inhibitors and combination therapies are crucial for improving cancer therapeutics and reducing mortality rates.

## Figures and Tables

**Table 1:** A summary of main kinases, phosphatases, and their substrates in DSBs repair in mammalians.

Subtract (Including Its Phosphorylated Form)	Kinase (In Vitro)	Phosphatase (In Vitro)
ATM	ATM [31,32], ATR [81], AurB [82], CDK5 [83], EGFR [84]	PPM1D [85] PPP2CA [86]
ATR	ATR [34], ABL [87], PKACA [88]	N/A
DNAPK	ATM [27], ATR [89], DNAPK [90], EGFR [91], PLK1 [92]	PPP6C [92]
CHK1	ATM [93], ATR [34], AKT1 [94], CDK1 [95], CHK1 [96], p90RSK [97]	PPM1D [69], PPP2CA [38]
CHK2	ATM [35], ATR [35], CHK2 [98], DNAPK [99], JAK2 [100], PLK1 [101], ZAK [102]	PPM1D [103], PPP1CA [103], PPP2CA [103]
H2AX	ATM [104], DNA-PK [105], CK2A1 [106], JMJD6 [107], MST1 [108], RSK2 [109]	EYA2 [110]. EYA3 [111], PPM1D [85,112], PPP2CA [113,114]
MDC1	ATM [115,116], ATR [117], CK2A1 [118], PLK1 [119,120]	N/A
53BP1	ATM [121], AMPKA1 [122], AMPKA2 [122], ATR [123], AurB [124], PLK1 [125]	N/A
RPA2	ATM [126], ATR [127,128], DNAPK [126,129,130]	PPP2CA [131], PPP4C [132]
Ku70/ Ku80	ATM [133,134], DNAPK [134], PKCA [135], Src [136]	N/A
NBS1	ATM [137,138,139,140,141,142], ATR [143], CDK2 [144]	PPP1CA [145]
Claspin	CDKL5 [146], CHEK1 [147], PLK1 [148]	N/A
BRCA1	Akt1 [149,150], ATM [141,151], ATR [152,153,154], AurA [155], CDK1 [156], CDK2 [157], CDK4 [158], CHEK2 [159], FRK [160], PLK1 [161]	PPP6C [162]
BRCA2	CDK1 [163], PLK1 [164,165]	N/A
P53	ATM [141,166,167,168,169,170,171,172,173,174,175,176,177,178,179], ATR [175,180], AurA [181,182,183], AurB [184,185], Btk [186], CDK1 [187,188,189], CDK4 [190], CDK5 [191], CDK6 [192,193], CDK9 [194], CHEK1 [195,196,197,198], CHEK2 [174,196,197,199], CK1A [200,201], CK2B [202], DAPK1 [197], DNAPK [195,196,197,203,204,205,206,207,208,209,210,211,212], DYRK1A [213], DYRK2 [214], ERK1 [215], ERK2 [216,217,218], GRK2 [219], GRK5 [220], HIPK2 [221,222], JNK1 [217,223], JNK2 [217], LKB1 [224], Lmr1 [225], LRRK2 [226], MAPKAPK5 [227,228], NEK2 [183], NuaK1 [229], P38A [227,230,231,232,233,234], P38G [233], PAK4 [235], PKCD [236], PKR [237], PLK3 [238,239,240], PRPK [241,242], SMG1, Src [243], TAF1 [244,245,246], VRK1 [247]	CDC14A [248], CDC25B [249], DUSP26 [250], PPM1D [85,251], PPP1CA [204], PPP2CA [252], PPP2CB [253], PPP2R5C [254]
MDM2	Abl [255,256], Akt1 [257,258,259,260,261,262], ATM [263,264,265], ATR [266], CDK9 [267], CK1D [268], ERK2 [269], MAPKAPK2 [270], PAK6 [271], Pim1 [272], PLK1 [273]	PPP2CA [274,275]
PLK1	Abl [276], AurA [277,278,279,280,281], CHEK1 [282], Cot [283], MAPKAPK2 [284], PAK1 [285], PLK1 [286], PLK2 [287], VRK2 [288]	PPP1CA [289]
WEE1	Akt1 [290], CDK1 [291]	CDC14A [291]
CDC25A	CDK1 [292,293,294], CDK2 [292] CHEK1 [295,296,297,298,299], CHEK2 [298,300], CK1A [301], DYRK2 [302], GSK3B [303], NEK11 [304], p90RSK [305], PLK3 [303], Src [294]	N/A
CDC25B	AurA [306], CDK1 [307], CHEK1 [308], MELK [309], p90RSK [305]	N/A
CDC25C	BRSK1 iso2 [310], CDK1 [311,312], CDK2 [313], CK2A1 [314], JNK1, JNK2 [315,316], PLK1 [317], TAK1 [318]	N/A
CDH1	CK1A [319], CK1E [319], PKCD [320], Src [321,322]	N/A

## Data Availability

Not applicable.
